# Uncertain behaviours of integrated circuits improve computational performance

**DOI:** 10.1038/srep16213

**Published:** 2015-11-20

**Authors:** Chihiro Yoshimura, Masanao Yamaoka, Masato Hayashi, Takuya Okuyama, Hidetaka Aoki, Ken-ichi Kawarabayashi, Hiroyuki Mizuno

**Affiliations:** 1 Center for Exploratory Research, Research & Development Group, Hitachi, Ltd., Kokubunji, Tokyo 185-8601, Japan; 2 Center for Technology Innovation-Information and Telecommunications, Research & Development Group, Hitachi, Ltd., Yokohama, Kanagawa 244-0817, Japan; 3 National Institute of Informatics, Chiyoda, Tokyo 101-8430, Japan; 4 Management Planning Office, Hitachi, Ltd., Chiyoda, Tokyo 100-8280, Japan

## Abstract

Improvements to the performance of conventional computers have mainly been achieved through semiconductor scaling; however, scaling is reaching its limitations. Natural phenomena, such as quantum superposition and stochastic resonance, have been introduced into new computing paradigms to improve performance beyond these limitations. Here, we explain that the uncertain behaviours of devices due to semiconductor scaling can improve the performance of computers. We prototyped an integrated circuit by performing a ground-state search of the Ising model. The bit errors of memory cell devices holding the current state of search occur probabilistically by inserting fluctuations into dynamic device characteristics, which will be actualised in the future to the chip. As a result, we observed more improvements in solution accuracy than that without fluctuations. Although the uncertain behaviours of devices had been intended to be eliminated in conventional devices, we demonstrate that uncertain behaviours has become the key to improving computational performance.

Von Neumann-architecture computers, which include all current mainstream computers, execute algorithms to solve problems. The performance of von Neumann-architecture computers has mainly been improved through semiconductor scaling^1^. However, improved performance will decelerate when semiconductor scaling ends^2^, and new computing paradigms emerge.

New computing paradigms have two characteristics: spatial representation of a problem and computer hardware that is analogous to the problem. The problems to be solved are represented spatially such as through neural networks and the Ising model. Neural networks are mimics of the brain that can implement machine learning and recognition^3^,^4^,^5^,^6^. Hardware implementations analogous to neural networks have been proposed and mainly implemented through silicon integrated circuits to achieve scalability in the number of neurons^7^,^8^,^9^,^10^. The Ising model is a statistical mechanics model of magnetism that was invented by Wilhelm Lenz in 1920^11^. The ground-state search of the Ising model, which means the determination of the spin configuration that minimises the energy function of the Ising model, is a kind of combinatorial optimisation problem and is essentially equivalent to the weighted maximum cut problem in graph theory^12^. The maximum cut problem is an archetype of the non-deterministic polynomial time hard (NP-hard) problem^13^, as is the ground-state search of the Ising model^14^. Finding the global optimum solution to the NP-hard problem generally needs exponential time. Approximation algorithms, which can find relatively better local optimum solutions, are used to solve the problem in practical time. Hardware implementations analogous to the Ising model have also been proposed and can achieve approximate solutions^15^,^16^,^17^,^18^,^19^.

We need to escape from local optima in both learning and optimisation. Simulated annealing, which is an optimisation algorithm inspired by the process of annealing in metallurgy, is widely used that probabilistically accepts state transitions to worse solutions according to the acceptance rate^20^. The acceptance rate is determined by the scheduled temperature and energy difference between the current state and worse state. Annealing is also used in neural networks called Boltzmann machines^21^. Probabilistic behaviours are achieved by comparison with random numbers generated by a pseudo-random number generator^22^,^23^. This means that generating random numbers is part of an algorithm, which must be accurately executed. However, computerised devices will exhibit uncertain behaviours due to semiconductor scaling. The cost, both in money and hardware resources, will be need to be increased to guarantee accurate behaviours in the near future. Algorithms that permit the existence of uncertainty have been proposed to improve energy efficiency in future semiconductor processes^24^.

Here, we demonstrate that solving optimisation by using uncertainty in devices is the source of randomness. More specifically, we carried out an experiment on the ground-state search of the Ising model by using a memory cell array with uncertainty that was implemented as a single silicon integrated circuit. We found that the uncertain behaviours of the hardware became usable as part of the algorithm.

## Results

### Escaping from local optimum by error

Local search is the foundation of optimisation algorithms including both simulated annealing and our method. The goal of optimisation is finding the solution, ***s***_*g*_, that minimises energy function *E*(***s***). We start from the initial state, ***s***_0_, in the local search and state transition occurs iteratively *N*-times ***s***_1_ … ***s***_*N*_. The neighbouring state, 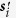, of the current, ***s***_*i*_, is generated in each step (*i*-th step), and next state ***s***_*i*+1_ is determined by:


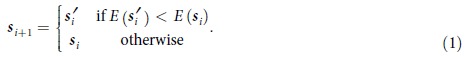


This behaviour can find a state that has energy lower than that of the current state. However, it is impossible to improve the solution once the state falls into a local optimum. This means the state transits to the same state forever (***s***_*i*+1_ = ***s***_*i*_).

Simulated annealing is a combination of the local search in Eq. (1) and the metropolis method^25^ with temperature control, and its behaviour is defined by:


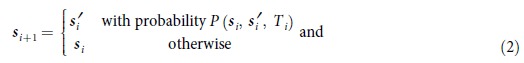



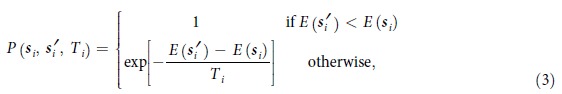


where *T*_*i*_ is temperature at the *i*-th step and *P* is the probability function. The state transition to the neighbouring state in Eq. (2) is accepted with probability *P*. When the neighbouring state has lower energy, the state transition is always accepted just like it is with local search in Eq. (1). However, simulated annealing even accepts state transition to a higher energy state with probability according to the energy difference and current temperature *T*_*i*_. Temperature is scheduled by:


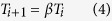


where *β* is the cooling coefficient (0 < *β* < 1). Temperature is exponentially decreased from given initial temperature *T*_0_.

A pseudo-random number generator is needed to achieve probabilistic behaviour as part of the algorithm. Threshold accepting^26^,^27^, which eliminates the necessity for randomness from simulated annealing, can be described by:


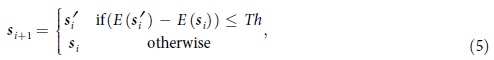


where *Th* is the threshold that takes the role of temperature *T* in simulated annealing. Threshold accepting still needs the energy difference between current and neighbouring states 
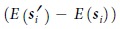
 to be calculated.

Here, we propose a method for the optimisation problem that does not need the energy difference to be calculated, where the bits representing the next state, ***s***_*i*+1_, are randomly flipped in agreement with the local search in Eq. (1). The probability of flipping the bits is described by:


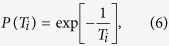


where *P* is the probability function and *T*_*i*_ is the temperature at the *i*-th step, as shown in Eq. (4). The behaviour of Eq. (6) is independent of local search, and it can be implemented as intentionally occurring error in the memory that stores state ***s***_*i*+1_.

### Ground-state search of Ising model

We applied the proposed method of the ground-state search of the Ising model. The *N*-spins Ising model can be described as the energy function:


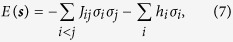


where ***s*** = {*σ*_1_, *σ*_2_, …, *σ*_*N*_} is a state of the Ising model called a spin configuration, *σ*_1_ = {+1, −1} is a spin, *J*_*ij*_ is an interaction coefficient between the *i*-th and *j*-th spins, and *h*_*i*_ is an external magnetic field for the *i*-th spin. The goal of the ground-state search is to find a spin configuration that minimises the energy function.

The local search of the Ising model can be achieved by using the nearest-neighbour interactions of individual spins. Each *i*-th spin is pulled in the direction of +1 or −1 by the force of the interaction between nearest-neighbour spins and the external magnetic field. The next state of an *i*-th spin that minimises the local energy within the neighbourhood can be determined by:


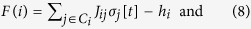



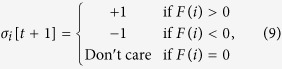


where *C*_*i*_ is the set of the nearest-neighbour spins for the *i*-th spin, *σ*_*j*_[*t*] is the current state of the *j*-th spin, 1 and *σ*_*i*_[*t* + 1] is the next state of the *i*-th spin at time *t*. Therefore, the next state of *i*-th spin *σ*_*i*_[*t* + 1] is determined so that the product of *i*-th spin *σ*_*i*_[*t* + 1] and *F*(*i*) becomes a positive value.

### Ising chip

**Figure 1:**
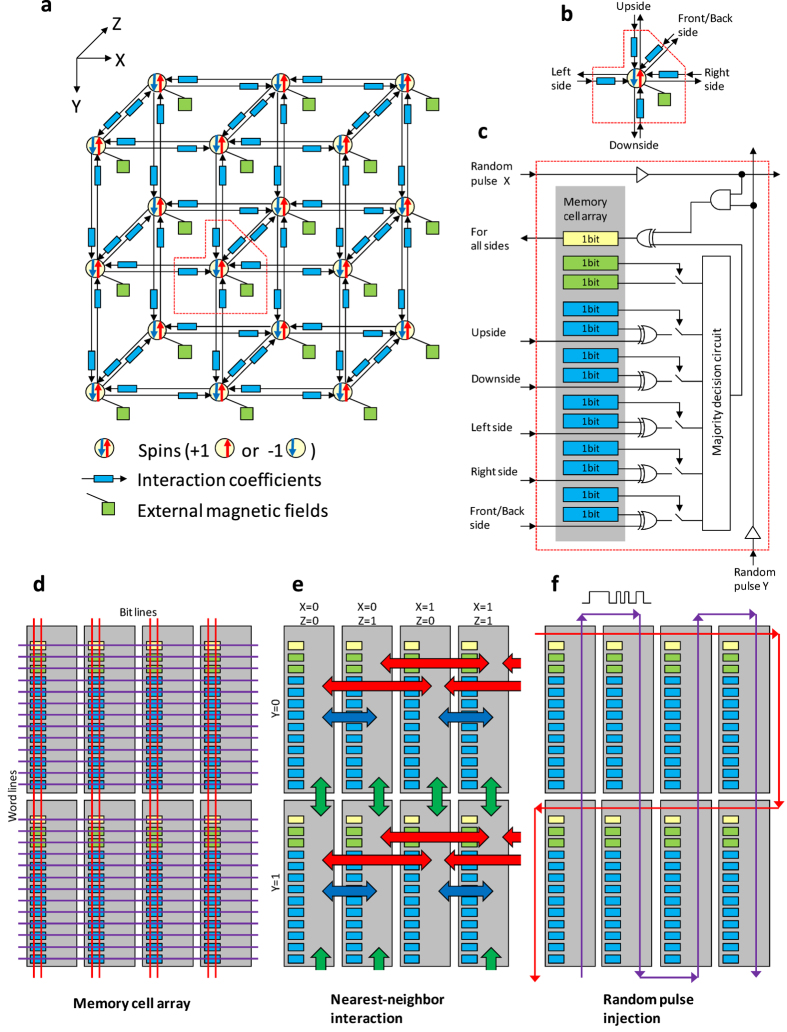
Mapping Ising model into array of processing elements called spin units. (**a**) Three-dimensional Ising model with directed interaction coefficient and external magnetic field. This model can be constructed with array of processing elements called spin units. (**b**) Spin unit that contains spin and coefficients affect spin. (**c**) Circuit diagram of spin unit. Spin unit has memory cells to represent spin and coefficients, and logic circuits to determine next state of spin for minimising energy. (**d**–**f**) Spin units are connected to three different wirings.

**Figure 2:**
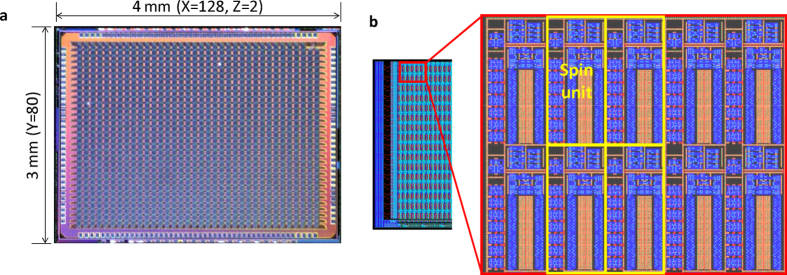
Single-silicon-memory-chip based Ising model solver: “Ising chip”. (**a**) Photograph of Ising chip fabricated with 65-nm bulk complementary-metal-oxide-semiconductor (CMOS) process. Chip size is 4 × 3 = 12 mm^2^ and contains 128 × 80 × 2 three-dimensional lattice Ising model. Z-axis is extracted into X-axis to embed three-dimensional lattice topology into planar chip. Thus, chip is 256 × 80 array of processing units called “spin units”. (**b**) Layout of spin unit on Ising chip.

The proposed method was implemented as the single silicon integrated circuit shown in Figs 1 and 2. A three dimensional lattice Ising model with free boundary conditions and 20-k (128 × 80 × 2) spins outlined in Fig. 1(a), whose ground-state search is an NP-hard problem, were implemented as a single silicon chip called an Ising chip^28^, which is shown in Fig. 2(a). The chip was constructed as a repetition of the unit element called a “Spin unit” to enable scalability. A spin and accompanying coefficients are grouped into a spin unit outlined in Fig. 1(b). A three dimensional lattice topology was extracted to the two dimensional array of spin units shown in Fig. 2(b). Each spin unit had a memory cell array to represent a spin and the coefficients outlined in Fig. 1(c). The next state of the spin was determined by the digital logic gate and analog majority decision circuit according to Eqs. (8) and (9).

The memory cells could be accessed via the bit lines and word lines outlined in Fig. 1(d) from the outside of the Ising chip in the same way as that in static random access memory (SRAM). The Ising chip had an inter-spin unit connection for local search and random bit flipping unlike a conventional memory chip. The spin units were connected as outlined in Fig. 1(e) according to the topology of the Ising model. The connections transferred the values of spins from nearest-neighbour spin units. All spin units were connected to the wires outlined in Fig. 1(f) that distributed random pulse signals. The random pulses emulated the uncertain behaviours of semiconductors in future processes. The memory cells representing the spins changed randomly being affected by the random pulses. The random pulses were injected from outside the Ising chip and the ratio of high and low was controlled to satisfy the probability of bit flipping shown in Eq. (6).

Comparison with the conventional computer, the Ising chip has several significant differences. The conventional computer is controlled by the program, which is sequence of the instruction for the central processing unit. All programs and data are stored in the main memory, and the central processing unit reads and writes the main mainly. The interconnection between the memory and the central processing unit leads performance bottleneck and power consumption. Unlike the conventional computer, the Ising chip is not controlled by the program. The Ising chip is a kind of analog computer that behavior is defined by hardware property. All data including spins and coefficients have been placed at the closest to the location to compute. The hardware structure is corresponding to the spatial structure of the problem. These features lead simple small hardware implementation with low power consumption (49.2 mW to do inter-spin interaction).

**Figure 3:**
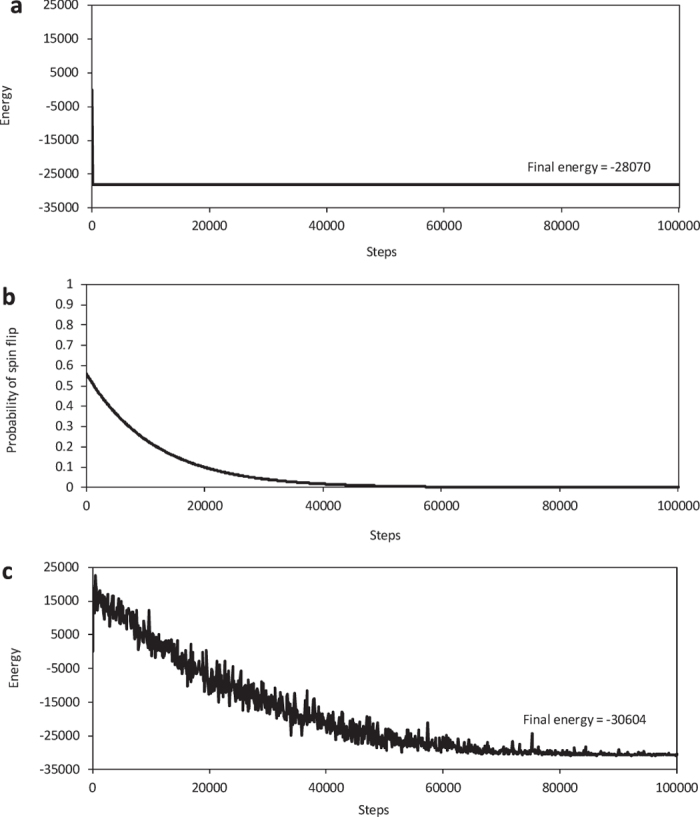
Process of ground-state search of Ising model by Ising chip. (**a**) Energy history of ground-state search without randomness to escape local optimum; spin configuration falls into local optimum through inter-spin interactions. (**b**) Schedule of probability of spin flips to escape from local optimum. (**c**) Energy history of ground-state search with randomness shown in (**b**) spin configuration reaches better solution.

**Figure 4:**
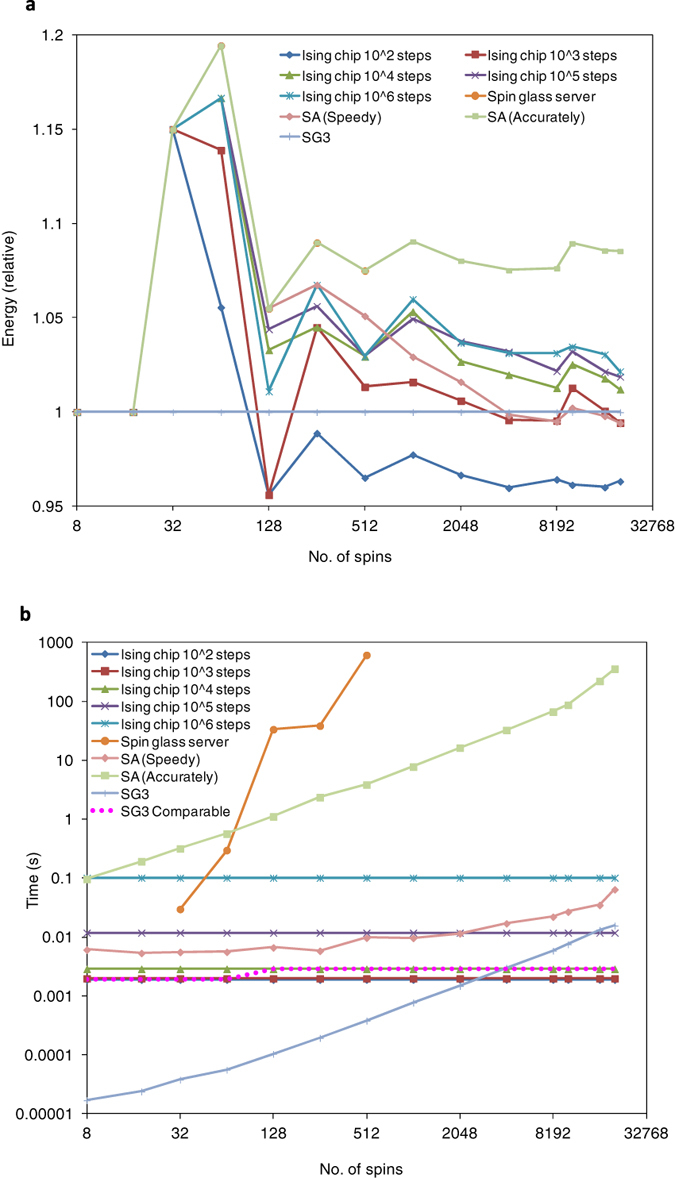
Performance evaluation of Ising chip. (**a**) Relationship between problem size (number of spins) and solution accuracy (relative value of energy). (**b**) Relationship between problem size and elapsed time to solve problem.

The results obtained from the ground-state search of Ising models by using the Ising chip are presented in Figs 3 and 4. The Ising models were generated randomly with various problem sizes under the restrictions of the specifications for the Ising chip. The topology of an Ising model is a three-dimensional lattice with free boundary conditions, as was previously described. This means spins have interaction coefficients in a lattice pattern. Each coefficient is randomly determined from two possible coefficients: +1 and −1. The ratio of coefficient r is also varied from *r* = 0 (all coefficients are −1) to *r* = 1 (all coefficients are +1).

Figure 3 plots the process for the ground-state search by using the Ising chip with or without random pulses for random bit flipping. One step is equivalent to 100 ns through operation of the Ising chip. There is a common problem in all experiments in Fig. 3 that had 20-k spins and *r* = 0.5. The state fell into a local optimum (at energy −28070) in the first several steps by only applying local search and the solution never improved as plotted in Fig. 3(a). Memory cells representing spins were randomly flipped by injecting random pulses to further improve the solution, and the probability of random flips is plotted in Fig. 3(b). The solution improved more than the previous solution as plotted in Fig. 3(c) by applying local search and random flips. The randomness helped to escape from the local optimum and the state could reach a better solution that was not possible with local search only.

Figure 4 plots performance evaluations of the Ising chip with various problem sizes. The ratio of coefficients is *r* = 0.5 in all the experiments in Fig. 4, which is the same as in the experiments in Fig. 3. Performance has two main aspects of accuracy and time. Time, which is needed to solve the problem, should be evaluated under conditions that can achieve at least the same accuracy. The Ising chip was evaluated with various numbers of steps such as 10^2^, 10^3^, 10^4^, 10^5^ and 10^6^. The number of steps was equivalent to the time to solve the problem, and the Ising chip had a tradeoff between the steps (time) and accuracy.

Figure 4(a) plots the accuracy of the solution with various problem sizes. The same problem is solved by using ten Ising chips and the best solution is selected. We evaluated the accuracy of the solution by comparing it with three algorithms on a conventional computer that had a Spin Glass Server^29^, SG3^30^ and simulated annealing. The Spin Glass Server is a cloud service to compute the exact ground-state of the Ising model to find the global optimum. The exact ground-state from the Spin Grass Server indicated an upper bound for accuracy. However, the problem size was limited to 512 spins. The SG3 is a greedy algorithm for the maximum cut problem of the graph and it is relatively faster because its computation time is almost proportional to the number of spins (or nodes in a graph). The maximum cut problem is essentially equivalent to the ground-state search of the Ising model without an external magnetic field as was previously explained. The SG3 can provide an approximate ground-state even with larger problem sizes. We have used the highly optimised implementation of simulated annealing for Ising model called optimised simulated annealing^31^. Optimised simulated annealing needs some parameter as same as Ising chip. We have used two parameter sets that are referred as SA (Speedy) and SA (Accurately). SA (Speedy) is the configuration that emphasises the computing time comparable to the Ising chip. SA (Accurately) spends a long period of computing time as much as possible in the experiment to emphasises the solution accuracy.

The quality of the solution can be measured by the energy that is calculated with the energy function. However, it is difficult to compare the quality with various problem sizes by using energy as a metric because the energy of the global optimum solution differs for each problem. Therefore, we defined the relative energy as a metric for comparison that is defined by:


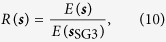


where ***s*** is the solution derived from the method for comparison, and ***s***_SG3_ is the solution to the same problem derived from the SG3 algorithm. The method for comparison can achieve better accuracy than SG3 when the relative energy is greater than one.

Figure 4(b) plots the computing times for the methods we described. The accuracy of the Ising chip depends on both the steps and problem size as indicated in Fig. 4 (a). The red dotted line (SG3 comparable) plots the computing time to achieve accuracy equal to or better than that of the SG3 algorithm. The number of steps for the ground-state search by the Ising chip was chosen appropriately for each problem size.

**Figure 5:**
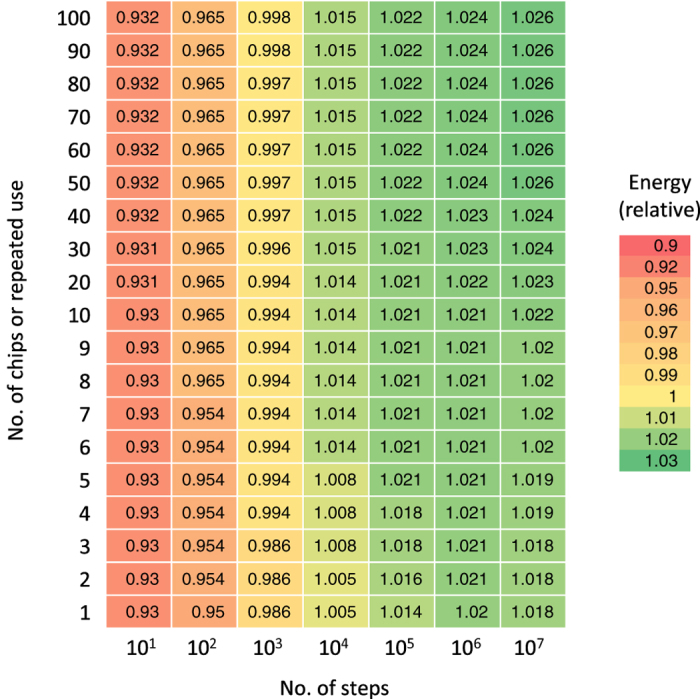
Solution accuracy of Ising chip in various configurations. Solution accuracy depends on number of steps in single ground-state search and number of iterations in repeating ground-state search.

The accuracy of the ground-state search depends on the number of steps. Plural Ising chips improve accuracy as was previously explained and ten Ising chips were used in the experiments. Figure 5 lists the accuracy achieved by combining the number of steps and the number of chips. This experiment used the 20-k spin problem with *r* = 0.5.

### Memory error under voltage control

However, the uncertain behaviours of miniaturised semiconductor devices in the future will be a serious problem for the conventional computer architecture. We demonstrated that the uncertain behaviours of transistors can be used as a source of randomness in each spin unit. The memory cells in an Ising chip represent spins and coefficients. The bit error rate (BER) of the memory cells in an SRAM is varied in the supply voltage^32^. Sufficiently high voltage (~1 V) is supplied to the SRAM in conventional computers to maintain accurate memory by using lower BER. We set the lower voltage (~0.7 V) to intentionally induce memory error in the SRAM. Furthermore, memory read operation, which is called dummy read, was executed during the period of lower voltage to increase the bit error rate so that it was higher than that without read operation.

**Figure 6:**
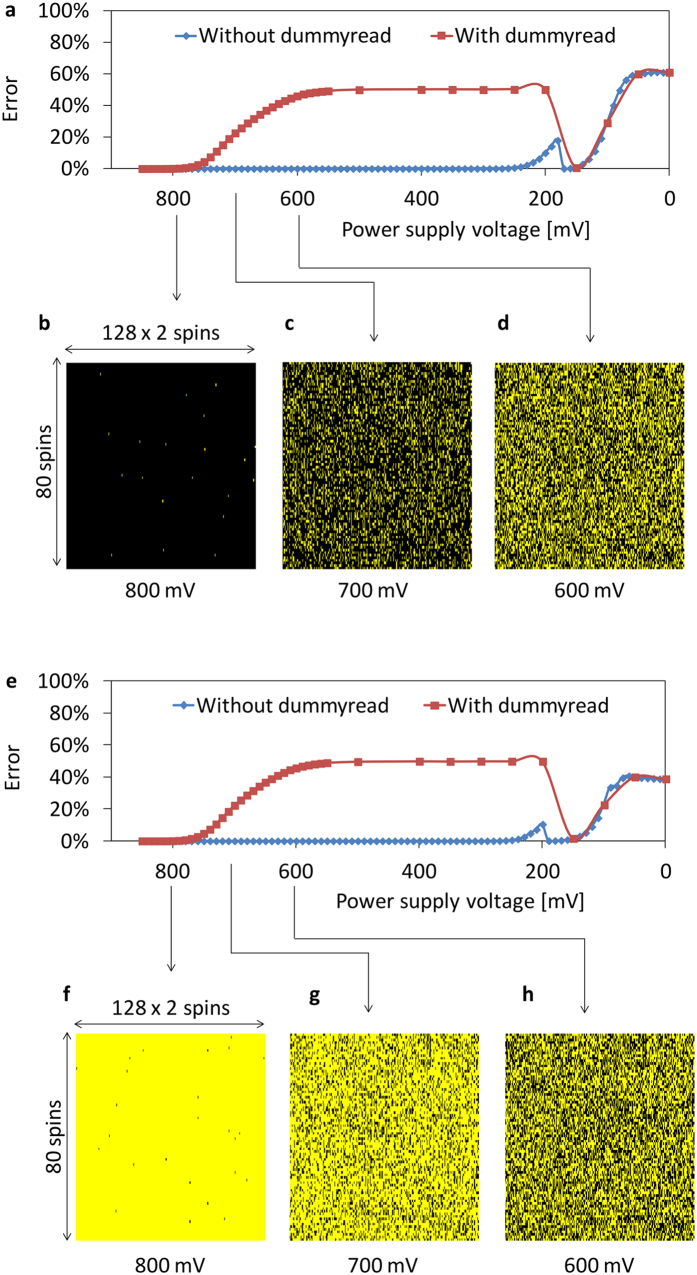
Randomness of memory cell values under voltage control. Bit error rate of memory cells of spins with various voltages. Initial value of zero is indicated in (**a**), and initial value of one is indicated in (**e**). Distributions of bit error on chip at 800 mV (**b**,**f**), 700 mV (**c**,**g**), and 600 mV (**d**,**h**) are shown. Black dots represent values of zero, and yellow dots represent values of one.

Figure 6 plots the randomness of memory cell values under voltage control and dummy read operation. We controlled the power supply voltage of memory cells that represented the value of spins. The memory cell value of zero represented spin state −1, and the memory cell value of one represented the spin state +1. In both kinds of initial values, zero and one, we can observe the spatially random pattern of memory cells according to the voltage. Dummy read operation accelerates the occurrence of bit errors and it achieves bit error in a relatively high voltage that is easier to control.

**Figure 7:**
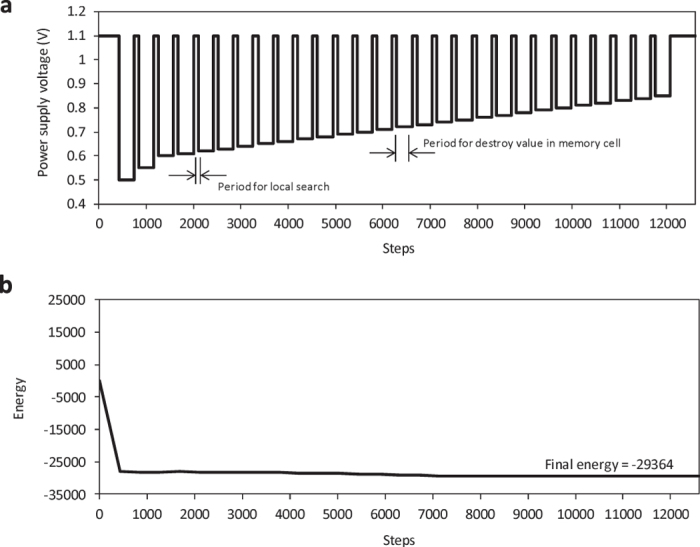
Ground-state search of Ising model by use of voltage-controlled memory cell. (**a**) Schedule of voltage control for power supply. (**b**) Energy history of ground-state search with voltage control to escape local optimum.

Figure 7 plots the ground-state search of the Ising model by using the previously mentioned voltage control scheme. Figure 7(a) presents the schedule of voltage control for power supply to the memory cells that represents the value of spins. This schedule corresponds to the probability of spin flips plotted in Fig. 7(b). Figure 7(b) plots the process of ground-state search with this methodology. The quality (energy) of the answer is better than that with local search plotted in Fig. 3(a), but worse than when using random pulses shown in Fig. 3(c). The cause of this phenomenon is that the randomness of memory cells is mainly dominated by static properties in the 65-nm node.

**Figure 8:**
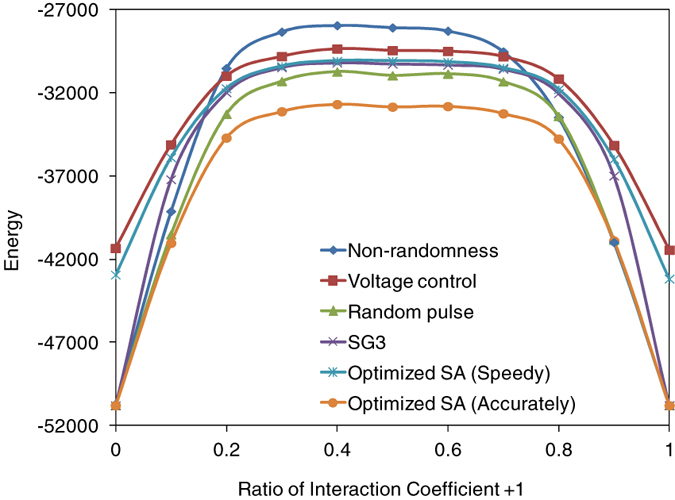
Comparison between previous algorithms and proposed methods. Previous algorithms and proposed methods are evaluated with various problems. Problems are varied by ratios of interaction coefficients +1 and −1.

Figure 8 plots the ground-state search of the various Ising models by using Ising chip and previous algorithms. The ratio of coefficients are varied from *r* = 0 to *r* = 1.0. All models are 20-k spin models. SA (speedy) and voltage controlled Ising chip has similar performance in the solution accuracy.

The time-independent fluctuations of transistors in the semiconductor chip, which are called mismatch properties, have been studied and their causes have been analysed^33^. One cause of mismatch is random dopant fluctuations (RDFs) that affect the threshold voltage of transistors^34^. The variations in threshold voltage are spatially random but temporally permanent. The fluctuations due to RDF are increased according to process miniaturisation and they determine the limits of scaling^35^. The effect by RDF should be suppressed under normal operation conditions from the viewpoint of SRAMs as memories in computers, and this can be done by optimising the device structure in current processes including the 65-nm node^36^. Time-independent spatial randomness from RDF is another viewpoint, and is used as a fingerprint to identify individual semiconductor chips^37^,^38^,^39^. However, few memory cells have time-dependent random behaviours and these are obstacles to fingerprints.

The temporal random behaviours of transistors and memory cells occur due to random telegraph noise (RTN)^40^,^41^,^42^. The influence of RTN has been increasing according to device scaling and its growth is faster than that of RDF^43^,^44^. The impact of RTN is expected to be more dominant than that of RDN in the 15-nm node. Temporal random behaviours by memory cells in SRAMs have been observed^45^,^46^ and bit errors have varied both in space and time. RTN is an obstacle to conventional usage. However, we expect that temporal variations in memory cell behaviours will help to search for better states in ground-state searches.

## Discussion

We examined the possibility of using fluctuations in device characteristics in this study as computational resources by carrying out experiments on real integrated circuits. We chose the optimisation problem, especially the ground-state search of the Ising model, as an example for the proof of concept. The randomness inherent in current devices was tested but that effect was insufficient for the ground-state search because temporally-static behaviours are dominant in current devices. The emulation of expected temporally-dynamic behaviours in future can achieve significant results, which would be comparable to the well-known greedy algorithm in conventional computers. We have proposed the use of random telegraph noise (RTN) as a source of randomness. However, the characteristics of RTN are still being investigated. The time constant of RTN and its controllability have become the main problem in applying the effect of RTN to information processing.

## Methods

### Detailed structure of chip in experiment

See Yamaoka *et al.*^28^ for the detailed structure of the chip. The chip was operated at 10 MHz clock frequency for the interactions.

### Generating problem for experiment

We generated various Ising models for the experiment. All problems were three-dimensional lattice Ising models without external magnetic coefficients. We had two aspects in the variations: problem size and the ratio of interaction coefficients. The variations in problem sizes were 8 (2 × 2 × 2) spins, 18 (3 × 3 × 2) spins, 32 (4 × 4 × 2) spins, 64 (8 × 4 × 2) spins, 128 (8 × 8 × 2) spins, 256 (16 × 8 × 2) spins, 1 k (16 × 16 × 2) spins, 2 k (32 × 16 × 2) spins, 4 k (32 × 32 × 2) spins, 8 k (64 × 32 × 2) spins, 10 k (64 × 64 × 2) spins, 16 k (128 × 64 × 2) spins, and 20 k (128 × 80 × 2) spins. The interaction coefficients of three-dimensional lattice topology were randomly chosen from {+1, −1} according to the ratio of interaction coefficient *r*.

### Random pulse injected to chip

Two random pulse signals were injected into the chip. The signals were generated by the pseudo-random number generator. The signals were generated three-times faster than the clock rate to operate the chip. The random pulse signals had high-level and low-level periods. The product of two signals was used in each spin inside the chip. The spin was flipped when the product of two signals was a high-level period. The probability of a high-level period occurring was defined as the mark rate. The scheduled spin flip plotted in Fig. 3(b) was achieved by dynamically changing the mark rate. Mark rate *p*(*t*) at time *t* is defined as:


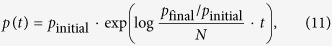


where *N* is the number of steps to solve the Ising model, *p*_initial_ is the initial mark rate, and *p*_final_ is the final mark rate at step *N*. After that, we add the period of the mark rate of zero to 1000 steps to stabilise the solution. The *p*_initial_ was 0.75 and *p*_final_ was 0.01 for all the experiments discussed in the paper. The random pulse signals were generated by comparison with the mark rates and random numbers. The random numbers were generated by the pseudo-random number generator^47^. The random signal is high-level when *r* < *p*(*t*), where *r* is the output from the pseudo-random number generator.

### Initial spin values

All the initial spin values were −1 in all the experiments described in this paper to align the experimental conditions.

### Previous algorithms for performance evaluation

In this paper, three implementations of previous algorithms, which is running on the conventional computers, have been used for the performance evaluation: Spin Glass Server^29^, SG3^30^ and optimized simulated annealing^31^. These implementations are available at refs 29,48,49 respectively. SG3^30^,^48^ has been executed at the popular personal computer (Intel Core i5 1.87 GHz, 8 GB Memory, Windows 7). It is enough resource for SG3 because the algorithm uses single core only. Optimized simulated annealing^31^,^49^ has been executed at the high-performance server (Hitachi HA8000/RS440, Intel Xeon E7-4807 1.87 GHz × 4 sockets, 256 GB Memory, CentOS 6.5). We have used “an_ms_r1_nf_omp” program of the optimized simulated annealing because it is highly optimized for the problem of our experiment. We have assumed two experiment conditions: SA (Speedy) and SA (Accurately). Speedy conditions are 10 sweeps and 10 repetitions (-s 10 -r 10 options are used). Accurately conditions are 10^4^ sweeps and 10^3^ repetitions (-s 10000 -r 1000 options are used). Sweeps means the number of updating each spin in one annealing process. Multiple annealing processes, which multiplicity is defined by repetitions parameter, are executed and the best solution is chosen. The “an_ms_r1_nf_omp” program uses the parallelism in the 64 bits integer number. Therefore, actual multiplicity is multiplied by 64.

## Additional Information

**How to cite this article**: Yoshimura, C. *et al.* Uncertain behaviours of integrated circuits improve computational performance. *Sci. Rep.* **5**, 16213; doi: 10.1038/srep16213 (2015).

## References

[b1] Markov L. Limits on fundamental limits to computation. Nature 512, 147–154 (2014). 25119233 10.1038/nature13570

[b2] Skotnicki T., Hutchby J. A., King T. J., Wong H. S. & Boeuf F. The end of CMOS scaling: toward the introduction of new materials and structural changes to improve MOSFET performance. IEEE Circuits and Devices Magazine 21, 16–26 (2005).

[b3] McCulloch W. S. & Pitts W. H. A logical calculus of the ideas immanent in nervous activity. Bull. Math. Biophys. 5, 115–133 (1943). 2185863

[b4] Rosenblatt F. The perceptron: A probabilistic model for information storage and organization in the brain. Psy. Rev. 65, 386–408 (1958). 10.1037/h0042519 13602029

[b5] Rumelhart D. E., Hinton G. E. & Williams R. J. Learning representations by back-propagating errors. Nature 323, 533–536 (1986).

[b6] Hinton G. E., Osindero S. & Teh Y. A fast learning algorithm for deep belief nets. Neural Computation 18, 1527–1554 (2006). 16764513 10.1162/neco.2006.18.7.1527

[b7] Merolla P. A. *et al.* A million spiking-neuron integrated circuit with a scalable communication network and interface. Science 345, 668–673 (2014). 25104385 10.1126/science.1254642

[b8] Schemmel J. *et al.* A wafer-scale neuromorphic hardware system for large-scale neural modeling. In Proc. of Int. Symp. Circuits and Systems 1947–1950 (IEEE, 2010).

[b9] Furber S. B., Galluppi F., Temple S. & Plana L. A. The SpiNNaker project. Proc. IEEE 102, 652–665 (2014).

[b10] Benjamin B. V. *et al.* Neurogrid: a mixed-analog-digital multichip system for large-scale neural simulations. Proc. IEEE 102, 699–716 (2014).

[b11] Brush S. G. History of the Lenz-Ising model. Rev. Mod. Phys. 39, 883–893 (1967).

[b12] Barahona F., Grötschel M., Jünger M. & Reinelt G. An application of combinatorial optimization to statistical physics and circuit layout design. Operations Research 36, 493–513 (1988).

[b13] Garey M. R. & Johnson D. S. Computers and Intractability: A Guide to the Theory of NP-Completeness (W.H. Freeman, New York, United States, 1979).

[b14] Barahona F. On the computational complexity of Ising spin glass models. J. Phys. A: Math. Gen. 15, 3241–3253 (1982).

[b15] Kastner M. A. Prospects for quantum dot implementation of adiabatic quantum computers for intractable problems. Proc. IEEE 93, 1765–1771 (2005).

[b16] Johnson M. W. *et al.* Quantum annealing with manufactured spins. Nature 473, 194–198 (2011). 21562559 10.1038/nature10012

[b17] Santra S., Quiroz G., Ver Steeg G. & Lidar D. A. MAX 2-SAT with up to 108 qubits. New J. of Phys. 16, 045006 (2014).

[b18] Utsunomiya S., Takata K. & Yamamoto Y. Mapping of Ising models onto injection-locked laser systems. Optics express 19, 18091–18108 (2011). 21935175 10.1364/OE.19.018091

[b19] Yoshimura C., Yamaoka M., Aoki H. & Mizuno H. Spatial computing architecture using randomness of memory cell stability under voltage control. In *Proc. European Conf. Circuit Theory and Design* (IEEE, 2013).

[b20] Kirkpatrick S., Gelatt C. D. & Vecchi M. P. Optimization by simulated annealing. Science 220, 671–680 (1983). 17813860 10.1126/science.220.4598.671

[b21] Ackley D. H., Geoffrey E. H. & Terrence J. S. A learning algorithm for Boltzmann machines. Cognitive science 9, 147–169 (1985).

[b22] Rutenbar R. A. Simulated annealing algorithms: An overview. IEEE Circuits and Devices Magazine 5, 19–26 (1989).

[b23] Johnson D. S., Aragon C. R., McGeoch L. A. & Schevon C. Optimization by simulated annealing: An experimental evaluation. Part II, Graph coloring and number partitioning. Operations research 39, 378–406 (1991).

[b24] Hamamoto M. & Yamaoka M. An energy-efficient parallel-processing method based on master-hibernating DVFS. In Proc. Int. Symp. on Circuits and Systems 1724–1727 (IEEE, 2014).

[b25] Metropolis N. *et al.* Equation of state calculations by fast computing machines. J. Chem. Phys. 21, 1087–1092 (1953).

[b26] Dueck G. & Scheuer T. Threshold accepting: A general purpose optimization algorithm appearing superior to simulated annealing. J. Comput. Phys. 90, 161–175 (1990).

[b27] Dueck G. New optimization heuristics: The great deluge algorithm and the record-to-record travel. J. Comput. Phys. 104, 86–92 (1993).

[b28] Yamaoka M. *et al.* 20-k-spin Ising chip for combinatorial optimization problem with CMOS annealing. ISSCC Dig. Tech. Papers 432–433 (IEEE, 2015).

[b29] Spin glass server. at < http://www.informatik.uni-koeln.de/spinglass/>.

[b30] Kahruman S., Kolotoglu E., Butenko S. & Hicks I. V. On greedy construction heuristics for the MAX-CUT problem. Int. J. Comput. Sci. Eng. 3, 211–218 (2007).

[b31] Isakov S. V., Zintchenko I. N., Rønnow T. F. & Troyer M. Optimised simulated annealing for Ising spin glasses. Computer Physics Communications 192, 265–271 (2015).

[b32] Kagiyama Y. *et al.* Bit error rate estimation in SRAM considering temperature fluctuation. In Proc. Int. Symp. Quality Electronic Design 516–519 (IEEE, 2012).

[b33] Pelgrom M. J., Duinmaijer A. C. & Welbers A. P. Matching properties of MOS transistors. IEEE J. of Solid-State Circuits 24, 1433–1439 (1989).

[b34] Stolk P. A., Widdershoven F. P. & Klaassen D. B. M. Modeling statistical dopant fluctuations in MOS transistors. IEEE Trans. on Electron Devices 45, 1960–1971 (1998).

[b35] Takeuchi K., Koh R. & Mogami T. A study of the threshold voltage variation for ultra-small bulk and SOI CMOS. IEEE Trans. on Electron Devices 48, 1995–2001 (2001).

[b36] Tachibana T. & Hiramoto T. Re-examination of impact of intrinsic dopant fluctuations on static RAM (SRAM) static noise margin. Jpn. J. Appl. Phys. 44, 2147–2151 (2005).

[b37] Lofstrom K., Daasch W. R. & Taylor D. IC identification circuit using device mismatch. ISSCC Dig. Tech. Papers 372–373 (IEEE, 2000).

[b38] Chellappa S., Dey A. & Clark L. T. Improved circuits for microchip identification using SRAM mismatch. In *Proc. Custom Integrated Circuits Conf.* 1–4 (IEEE, 2011).

[b39] Fujiwara H. *et al.* A stable chip-ID generating physical uncloneable function using random address errors in SRAM. In *Proc. Int. System-on-Chip Conf.* 143–147 (IEEE, 2012).

[b40] Agostinelli M. *et al.* Erratic fluctuations of SRAM cache Vmin at the 90nm process technology node. In IEDM Technical Digest 655–658 (IEEE, 2005).

[b41] Miki H. *et al.* Understanding short-term BTI behavior through comprehensive observation of gate-voltage dependence of RTN in highly scaled high-κ/metal-gate pFETs. In Symp. on VLSI Tech. 148–149 (IEEE, 2011).

[b42] Toh S. O., Liu T. J. K. & Nikolić B. Impact of random telegraph signaling noise on SRAM stability. In Symp. on VLSI Tech. 204–205 (IEEE, 2011).

[b43] Tega N. *et al.* Reduction of random telegraph noise in high-к/metal-gate stacks for 22 nm generation FETs. In IEDM Technical Digest 771–774 (IEEE, 2009).

[b44] Tega N. *et al.* Impact of HK/MG stacks and future device scaling on RTN. In Proc. of Int. Reliability Physics Symp. 6A.5.1–6A.5.6 (IEEE, 2011).

[b45] Takeuchi K. *et al.* Direct observation of RTN-induced SRAM failure by accelerated testing and its application to product reliability assessment. In Symp. on VLSI Tech. 189–190 (IEEE, 2010).

[b46] Takeuchi K., Nagumo T. & Hase T. Comprehensive SRAM design methodology for RTN reliability. In Symp. on VLSI Tech. 130–131 (IEEE, 2011).

[b47] Matsumoto M. & Nishimura T. Mersenne twister: A 623-dimensionally equidistributed uniform pseudo-random number generator. ACM Trans. on Modeling and Computer Simulation 8, 3–30 (1998).

[b48] Maehara T. Maximum cut solver. (2013). at < https://gist.github.com/spaghetti-source/7780280>.

[b49] Isakov S. V., Zintchenko I. N., Rønnow T. F. & Troyer M. Optimized simulated annealing for Ising spin glasses. (2014). at < http://arxiv.org/src/1401.1084v3/anc>.

